# An Investigation of the Etiology and Pattern of Spinal Injuries Among Patients Presenting to the Emergency Department in a Tertiary Care Hospital

**DOI:** 10.7759/cureus.112798

**Published:** 2026-07-16

**Authors:** Akshay Patil, Mandar Deshpande, Saurabh B Wasule, Dhruva Reddy, Dattatray Bhusare, Sagar Sinha, Shruti Pataki, Jayvardhan Lade, Shruti Menon, Pratik Chudiwal

**Affiliations:** 1 Department of Emergency Medicine, Mahatma Gandhi Mission Medical College and Research Institute, Navi Mumbai, IND

**Keywords:** emergency department, falls, neurological deficit, rta, spinal injury

## Abstract

Background

Spinal cord injuries contribute substantially to morbidity and mortality globally. There is a gross disparity in the causes of spinal injuries between developed and developing countries. Thus, understanding the etiology and pattern of spinal injury in our setting is important to plan strategies for effective management and prevention strategies at the national level.

Methodology

A prospective observational study was conducted among 75 patients with spinal cord injury visiting the Department of Emergency Medicine of a tertiary care hospital. The study aimed to investigate the etiology, clinical profile, radiological features, neurological outcomes, and short-term prognosis of patients presenting with acute spinal injuries, focusing on both traumatic and non-traumatic etiologies. A structured questionnaire was used to collect patient information and document the clinical findings. X-ray and CT spine were also performed to understand the type of injury.

Results

The mean age of 75 patients with spinal injury was 36.4 ± 8.7 years, with 41 (54.7%) males. Road traffic accident was the most common cause seen among 37 (49.3%) cases of injury, followed by falls among 20 (26.7%) cases. Based on the Canadian C-spine rule, a high-risk mechanism of injury was seen among 67 (89.3%) cases. Cervical spine injury was seen among 30 (40.0%) cases, while thoracic and lumbar spines were involved in 26 (34.7%) and 19 (25.3%) cases, respectively. The common neurological presentation was bilateral lower limb weakness, reported in 27 (36%) cases, with combined upper and lower limb weakness seen in 16 (21.3%), and quadriplegia occurred in 6 (8%) cases. Of the 75 patients, 42 (56%) underwent surgical treatment, while 33 (44%) were managed conservatively. Mortality was seen among 20 (26.7%) cases. Common complications included pressure ulcers in 11 (14.7%) cases, with 10 (13.3%) cases each of deep vein thrombosis, urinary tract infection, and pneumonia.

Conclusions

Spinal trauma was predominantly caused by road traffic accidents and falls. A significant proportion presented with neurological deficits, most commonly incomplete injuries. Surgical management demonstrated neurological improvement in selected cases. The study emphasizes the importance of early mobilization and the need for multidisciplinary care.

## Introduction

Spinal injuries are a major cause of morbidity and mortality worldwide and constitute a significant public health challenge [1]. The etiology of spinal injuries varies across different populations and regions, with the most common causes being road traffic accidents (RTAs), falls, hanging, sports-related injuries, violence, and occupational hazards. Early recognition, prompt diagnosis, and timely management of spinal trauma are essential to prevent secondary neurological damage, reduce long-term disability, and improve functional outcomes. Appropriate initial assessment, rapid immobilization, and the judicious use of diagnostic imaging play a pivotal role in optimizing patient care and reducing the burden of spinal injuries [2].

The epidemiology of spinal injuries varies considerably across the world, with differences in incidence, mechanisms, and patterns of injury influenced by demographic, socioeconomic, occupational, and environmental factors [3]. In high-income countries, RTAs remain the leading cause of spinal trauma, whereas in low- and middle-income countries, the burden is shared among RTAs, falls from height, occupational injuries, interpersonal violence, and other trauma-related events. These variations reflect differences in infrastructure, workplace safety, healthcare access, and injury prevention strategies. Young adult males are disproportionately affected because of their greater involvement in high-risk occupations, motor vehicle use, contact sports, and risk-taking behaviors. In contrast, older adults are more susceptible to spinal injuries due to age-related degenerative changes, osteoporosis, impaired balance, and an increased risk of falls [4]. Understanding these epidemiological variations is essential for developing targeted prevention strategies, optimizing trauma care, and reducing the burden of spinal injuries across different populations [1].

Delayed diagnosis or inappropriate initial management can lead to secondary spinal cord injury, permanent neurological impairment, and increased morbidity. Therefore, early recognition, prompt immobilization, and appropriate imaging are essential components of emergency care. Management of spinal injuries is guided by the type, stability, and severity of the injury, as well as the patient’s neurological status. Stable fractures without neurological compromise are generally managed conservatively using cervical collars, spinal braces, or traction, along with close clinical monitoring and rehabilitation. In contrast, unstable fractures, spinal instability, spinal cord compression, or progressive neurological deterioration require timely surgical intervention to achieve decompression, stabilization, and optimal neurological recovery [5]. A multidisciplinary approach involving emergency physicians, spine surgeons, radiologists, and rehabilitation specialists is crucial for improving functional outcomes and quality of life.

The emergency department (ED) serves as the first point of contact for most patients with spinal trauma and plays a pivotal role in the early recognition, stabilization, and management of these potentially life-threatening injuries. Prompt assessment and timely intervention in the ED are essential to prevent secondary spinal cord damage and improve neurological outcomes. The present study was undertaken to evaluate the etiology and pattern of spinal injuries among patients presenting to a tertiary care hospital. By examining the demographic profile, mechanisms of injury, anatomical distribution, clinical presentation, and outcomes of spinal trauma, the study provides valuable insights into the local epidemiology and burden of disease. Furthermore, it helps identify high-risk populations, common mechanisms of injury, and factors associated with morbidity and mortality. The findings of this study are expected to support evidence-based decision-making, optimize the utilization of diagnostic imaging, strengthen ED protocols, and guide the development of targeted injury prevention strategies.

## Materials and methods

Study design

A prospective observational study was conducted in the Department of Emergency Medicine, MGM Medical College and Hospital, Navi Mumbai, from August 2023 to March 2025. The study aimed to investigate the etiology, clinical profile, radiological features, neurological outcomes, and short-term prognosis of patients presenting with acute spinal injuries, focusing on both traumatic and non-traumatic etiologies.

Study population

The study included patients presenting to the ED with spinal injury and meeting the selection criteria. Patients were recruited upon confirmation of spinal injury based on clinical and radiological findings. Informed consent was obtained from all participants or their legal representatives.

Inclusion and exclusion criteria

Patients radiologically diagnosed with spinal injury and aged more than six years who provided consent were included in the study. The study excluded pregnant women, those with intellectual disabilities, and seriously/terminally ill patients. Patients for whom radiological imaging was not possible and those unwilling to participate were also excluded.

Sample size and sampling method

All spinal injuries presenting at the ED during the study period were included. A total of 82 patients fulfilled the inclusion criteria, of whom seven patients were excluded due to being terminally ill or the inability to perform radiological examination. Hence, the final sample was 75.

Ethical considerations

Ethical clearance was obtained from the Institutional Ethical Committee of MGM Medical College and Hospital (approval number: DHR-EC/SC/2023/07/18). Written informed consent was obtained from patients who were conscious, while for those who were unconscious, it was obtained from a family member. Confidentiality of patient data was maintained throughout the study.

Data collection procedure

Each enrolled patient underwent a structured evaluation using a semi-structured questionnaire which included the details of the demographic status (age, sex, occupation, residential background), mode and mechanism of injury, clinical assessment (vital signs, general examination, neurological status grading using American Spinal Injury Association (ASIA) Impairment Scale (AIS) [6], and associated injuries including head injury, chest trauma, limb fractures), radiological evaluation (plain X-rays of the spine, CT scans for vertebral fractures, retropulsion, and canal compromise). The Canadian C-spine rule (CCR) [7] was used to classify injury as a high-risk injury requiring radiological investigation of the cervical spine. Treatment modalities were recorded as surgical interventions (decompression, stabilization, spinal fixation, and fusion) and conservative management given (bracing, bed rest, and physiotherapy assessment).

Outcome measures

The primary outcome of the study included the level and type of spinal injury (cervical, thoracic, lumbar, and sacral), radiological patterns (wedge compression, burst fracture, dislocation, hematoma, and canal stenosis), neurological deficit grading (based on the ASIA scale on admission and follow-up), and complications, including pressure ulcers, deep vein thrombosis, pneumonia, and urinary infections. The secondary outcome was mortality and residual neurological deficit at 28 days.

Definition of neurological status and criteria for neurological improvement in spinal injury

In the context of spinal injury management, the assessment of neurological status is a critical determinant of patient outcomes. Neurological status refers to the functional state of the nervous system, particularly focusing on motor power, sensory perception, and reflexes, as evaluated in patients with spinal injuries. It is an essential parameter that directly influences clinical decision-making, rehabilitation planning, and prognosis assessment.

Neurological status assessment

Neurological status in patients with spinal injuries is typically assessed using standardized scales and clinical examination protocols. The AIS is widely used for this purpose, providing a comprehensive assessment of both motor and sensory functions. In addition to ASIA grading, a detailed examination of individual muscle groups for motor strength, dermatomal regions for sensory perception, and reflex arcs for integrity was performed.

Classification of neurological status in spinal injury

The neurological status of patients with spinal injury in this study was categorized into three primary outcomes (improved, worsened, or no change) based on changes in motor and sensory functions during the study period. Improved neurological status was defined as a measurable improvement in motor power or the presence of new sensory impulses in regions previously identified as impaired. Motor improvement was assessed by an increase of at least one grade on the Medical Research Council (MRC) scale for muscle strength, which ranges from 0 (no contraction) to 5 (normal strength) [8]. Sensory improvement was identified by the restoration of light touch, pain, or temperature sensation in previously affected dermatomes. Worsened neurological status was defined as a decrease in motor power or the complete loss of previously present sensory impulses. Motor deterioration was marked by a reduction of one or more grades on the MRC scale for muscle strength. Sensory loss was characterized by the absence of previously intact sensory modalities (light touch, pain, temperature) in the affected region. No change in neurological status was defined when the patient’s neurological condition remained unchanged from the initial assessment. There was neither an improvement in motor or sensory functions nor any deterioration.

Method of neurological assessment

Motor power assessment for each key muscle group associated with the thoracic, lumbar, and sacral spinal segments is evaluated using the MRC grading scale [8] (Table 1). Sensory assessment was evaluated in the dermatomal regions corresponding to the affected spinal segments, using standardized techniques [9] (Table 2).

**Table 1:** Medical Research Council (MRC) scale for muscle strength.

Grade	On examination
Grade 0	No contraction
Grade 1	Flicker or trace of contraction
Grade 2	Active movement with gravity eliminated
Grade 3	Active movement against gravity
Grade 4	Active movement against resistance
Grade 5	Normal strength

**Table 2:** Standardized technique for sensory assessment.

Sense examined	Assessment method
Light touch	Assessed using a cotton swab or fingertip
Temperature	Assessed using cold and warm objects
Vibration	Assessed using a tuning fork (128 Hz)
Proprioception	Assessed by passive movement of the patient’s limb and asking for recognition of the position
Deep tendon reflexes	Reflex testing is also performed for the evaluation of upper and lower limb reflexes (biceps, triceps, patellar, and Achilles reflexes)

Recording and documentation of neurological status

Neurological status was assessed at the time of admission, after initial stabilization, and subsequently on day 28. Changes in neurological status were meticulously documented in the patient’s case record form, with separate columns for motor strength, sensory perception, and reflex integrity. In cases where motor or sensory improvement was detected, specific dermatomes and muscle groups showing improvement were recorded. For patients with worsened neurological status, the affected regions and the extent of deterioration were clearly specified.

Statistical analysis

Data were compiled in Microsoft Excel (Microsoft Corp., Redmond, WA, USA) and analyzed using SPSS version 26.0 (IBM Corp., Armonk, NY, USA). Descriptive statistics were expressed as frequencies, percentages, mean, and standard deviation (SD). The chi-square test was used to detect the association between risk factors leading to mortality, with a p-value <0.05 considered to be statistically significant.

## Results

The mean age of patients with spine injury in the present study was 36.4 ± 8.7 years, with most belonging to the age group of 21 to 40 years. The majority were males (41, 54.7%). The occupational status of the patients is presented in Table 3.

**Table 3:** Sociodemographic details of the study participants (N = 75).

Variable	Frequency	Percentage
Age (in years)		
≤20	3	4.0
21–40	27	36.0
41–60	25	33.3
>60	20	26.7
Sex		
Female	34	45.3
Male	41	54.7
Occupation		
Driver	12	16.0
Housewife	18	24.0
Retired	15	20.0
Laborer	17	22.6
Student	5	6.7
Others	8	10.6

Based on the mode of injury, RTA was the most commonly seen among 37 (49.3%) cases, followed by falls among 20 (27.7%) cases. On classifying the mechanism of injury, 67 (89.3%) had a high-risk mechanism that required radiography of the cervical spine as per the CCR (Table 4).

**Table 4:** Mode and mechanism of injury (N = 75). *: High-risk injury needing cervical radiography was based on the Canadian C-spine rule [7].

Variable	Frequency	Percentage
Mode of injury		
Road traffic accident	37	49.3
Fall	20	27.7
Assault	4	5.3
Hanging	5	6.7
Sports	4	5.3
Others	5	6.7
Mechanism of injury*		
Radiography needed	67	89.3
Radiography not needed	8	10.7

In this study, cervical spine injury was seen among 30 (40.0%) patients, while thoracic and lumbar spines were involved in 26 (34.7%) patients and 19 (25.3%) patients, respectively. The level of spinal cord injury is detailed in Table 5. The most common neurological presentation was bilateral lower limb weakness, reported in 36% (27 patients). Combined upper and lower limb weakness was seen in 21.3% (16 patients), and quadriplegia occurred in 8% (6 patients). No neurological deficit was found in 18.7% (14 patients). Other less frequent findings included isolated sensory levels at specific spinal segments and complete loss of sensation below the lumbosacral junction in one patient, indicating a wide clinical spectrum from intact function to complete motor sensory loss.

**Table 5:** Distribution as per part of spine and predominant level of spinal injury involved (N = 75).

Level of spine injury	Frequency	Percentage
Cervical		
C2	3	4.0
C3	2	2.7
C4	7	9.3
C5	7	9.3
C6	4	5.3
C7	7	9.3
Thoracic		
T2	3	4.0
T3	1	1.3
T4	1	1.3
T6	1	1.3
T7	6	8.0
T8	3	4.0
T10	9	12.0
T12	2	2.7
Lumbar		
L1	7	9.3
L2	3	4.0
L3	3	4.0
L5	6	8.0

**Figure 1:**
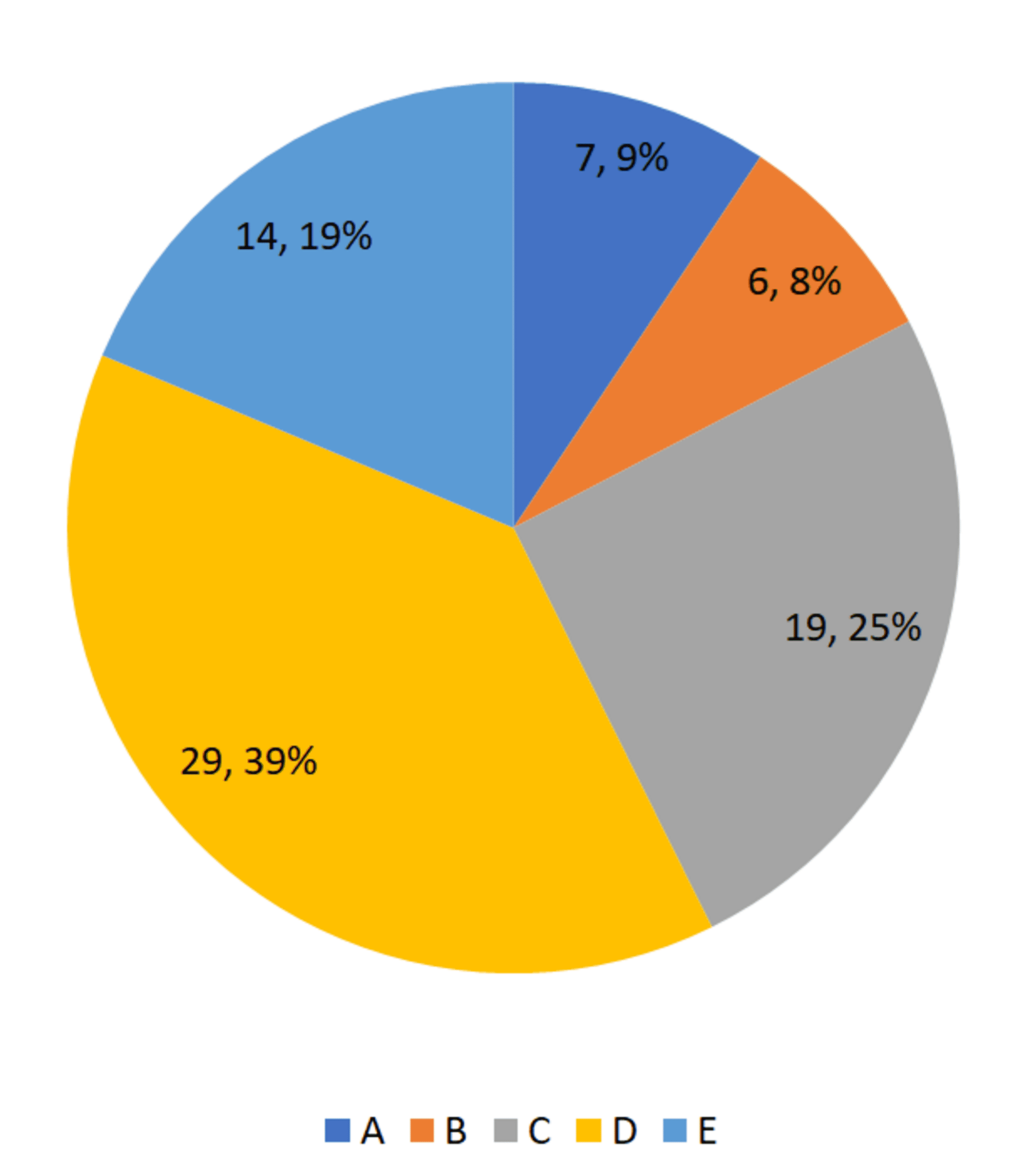
Distribution as per the American Spinal Injury Association scale score (N = 75).

Assessment via the ASIA scale showed that most patients were graded D (38.6%, 29 patients) and C (25.3%, 19 patients), indicating incomplete injury with preserved motor function. Grades A (complete injury) and B were seen in 9.3% (7 patients) and 8% (6 patients), respectively. Grade E, representing normal function, was seen in 18.7% (14 patients) (Figure 1).

The most common primary radiographic finding was wedge compression fractures seen among 28 (37.3%) patients, followed by dislocations in 19 (25.3%) patients. Other X-ray findings are presented in Table 6.

**Table 6:** Primary injury as per X-ray (N = 75).

X-ray finding	Frequency	Percentage
Burst	14	18.7
Compression	8	10.7
Dislocation	19	25.3
Fracture	6	8.0
Wedge	28	37.3

On CT imaging, additional findings included canal stenosis (24.0%, 18 patients), retropulsion (21.3%, 16 patients), and hematoma (26.7%, 20 patients). Other abnormalities are presented in Table 7.

**Table 7:** Distribution of patients as per additional CT spine findings.

CT spine findings	Frequency	Percentage
Canal stenosis	18	24.0
Fracture dislocation	10	13.3
Hematoma	20	26.7
Anterolisthesis	4	5.3
Retrolisthesis	4	5.3
Retropulsion	16	21.3
Transection	3	4.0

Of the 75 patients, 56% (42 patients) underwent surgical treatment, while 44% (33 patients) were managed conservatively, reflecting balanced use of both modalities depending on severity and stability. Among those requiring surgical management, fixation was done in 17 (40.5%) cases, decompression and fusion were each performed in 13 (31%) and 12 (28.5%) cases, respectively. There was no significant difference in the outcome among those receiving surgical and conservative treatment (p > 0.05) (Table 8).

**Table 8:** Treatment outcome at the end of seven days (N = 75).

Treatment	Outcome at seven days			Chi-square (p-value)
	No change	Improved	Worsened	
Surgical (n = 42)	20 (47.6%)	17 (40.5%)	5 (11.9%)	5.90 (0.052)
Conservative (n = 33)	21 (63.6%)	5 (15.2%)	7 (21.2%)	

The neurological outcome was classified using AIS criteria on days 7, 14, and 28. Those with Grade A showed the maximum proportion of mortality, i.e., 4 out of 7 (57.1%), while Grade E had the least mortality, i.e., 2 out of 14 (14.2%). Worsening of symptoms beyond 7 days was not seen in any grade except for one person from Grade D. Overall, 20 (26.7%) patients died during the follow-up on 28 days (Table 9).

**Table 9:** Neurological outcome of patients assessed at follow-up on day 7, 14, and 28. AIS = American Spinal Injury Association Impairment Scale

AIS on admission	Follow-up	Grade A	Grade B	Grade C	Grade D	Grade E	Death	Lost to follow-up
Grade A (n = 7)	Day 7 (n = 7)	5	1	0	0	0	1	0
	Day 14 (n = 6)	2	1	0	0	0	3	0
	Day 28 (n = 3)	2	0	0	0	0	0	1
Grade B (n = 6)	Day 7 (n = 6)	1	2	1	0	0	2	0
	Day 14 (n = 3)	0	1	0	0	0	1	1
	Day 28 (n = 2)	0	0	0	0	0	1	1
Grade C (n = 19)	Day 7 (n = 17)	0	0	10	4	1	2	0
	Day 14 (n = 15)	0	0	7	3	2	1	2
	Day 28 (n = 12)	0	0	7	2	2	0	1
Grade D (n = 29)	Day 7 (n = 29)	0	0	2	9	11	5	2
	Day 14 (n = 22)	0	0	1	8	10	2	1
	Day 28 (n = 17)	0	0	0	7	9	0	1
Grade E (n = 14)	Day 7 (n = 29)	0	0	1	3	23	1	1
	Day 14 (n = 27)	0	0	0	2	24	1	0
	Day 28 (n = 26)	0	0	0	2	24	0	0

In-hospital complications included pressure ulcers, which were noted among 11 (14.7%) patients, deep vein thrombosis, pneumonia, and urinary tract infection, seen among 10 (13.3%) cases each, while no complication was detected among 20 (26.7%) patients. Out of 75 patients, 20 (26.7%) succumbed during treatment, while 55 (73.3%) survived, indicating a significant mortality burden associated with spinal trauma in this setting.

Age more than 40 years, level of injury at the cervical level, ASIA scale score, and the presence of complications were found to be significantly associated with mortality (Table 10).

**Table 10:** Factors associated with mortality. RTA = road traffic accident; ASIA = American Spinal Injury Association

Variable		Mortality		Chi-square	P-value
		No (n = 55)	Yes (n = 20)		
Gender	Male (n = 41)	31 (75.6%)	10 (24.4%)	0.240	0.624
	Female (n = 34)	24 (70.6%)	10 (29.4%)		
Age	<40 years (n = 30)	29 (96.7%)	1 (3.3%)	13.92	0.001*
	≥40 years (n = 45)	26 (57.8%)	19 (42.2%)		
Mode of injury	Fall (n = 20)	14 (70.0%)	6 (30.0%)	2.96	0.226
	RTA (n = 37)	25 (67.6%)	12 (32.4%)		
	Others (n = 180	16 (80.0%)	2 (20.0%)		
Injury level	Cervical (n = 30)	15 (50.0%)	15 (50.0%)	13.94	0.001*
	Lumbar (n = 20)	18 (90.0%)	2 (10.0%)		
	Thoracic (n = 25)	22 (88.0%)	3 (12.0%)		
ASIA scale score	A (n = 7)	1 (14.3%)	6 (85.7%)	41.28	0.001*
	B (n = 6)	2 (33.3%)	4 (66.4%)		
	C (n = 19)	10 (52.6%)	9 (47.4%)		
	D (n = 28)	27 (96.4%)	1 (3.6%)		
	E (n = 15)	15 (100.0%)	0 (0)		
Associated injury	Present (n = 37)	26 (70.3%)	11 (29.7%)	0.350	0.554
	Absent (n = 38)	29 (76.3%)	9 (23.7%)		
Radiological imaging	Needed (n = 66)	46 (69.7%)	20 (30.3%)	3.710	0.054
	Not needed (n = 9)	9 (100.0%)	0 (0)		
Complication	Yes (n = 55)	37 (67.3%)	18 (32.7%)	3.870	0.049*
	No (n = 20)	18 (90.0%)	2 (10.0%)		

## Discussion

The present study described the causes and patterns of spinal injuries among patients presenting to the emergency department of a tertiary care hospital between August 2023 and March 2025. The mean age of the study participants was 36.4 ± 8.7 years, with a significant proportion of patients being middle-aged and older adults. Similar age distributions have been reported in studies conducted internationally [10,11,12] as well as in India [13]. The consistency of these findings across different geographic regions suggests that spinal trauma predominantly affects individuals in their economically productive years. This may be attributed to increased occupational exposure, greater involvement in road traffic, and higher levels of physical activity among this age group. These observations highlight the need for targeted preventive interventions, including workplace safety measures, road traffic injury prevention strategies, and public awareness programs aimed at reducing the burden of spinal injuries in the economically active population.

In the present study, males accounted for 54.7% of spinal injury cases, while females constituted 45.3%, indicating a modest male predominance. This finding is consistent with previous studies on spinal trauma, which have reported a higher proportion of male patients, ranging from 76% to 81% [10,11,13]. The predominance of males has been attributed to greater exposure to outdoor activities, occupational hazards, and an increased likelihood of involvement in RTAs. However, compared with these earlier reports, our study demonstrated a more balanced gender distribution. This difference may reflect evolving societal and occupational roles, increased mobility and workforce participation among women, and improved access to emergency healthcare services in urban settings. It may also indicate changing patterns of injury risk across both sexes, highlighting the need for preventive strategies that address spinal trauma in both male and female populations.

In the present study, RTAs were the leading cause of spinal injuries, accounting for 49.3% of cases, followed by falls, which constituted 22.7%. This pattern reflects a dual burden of high-energy trauma related to motor vehicle collisions and low-energy trauma resulting from falls. Similar findings have been reported in several studies where RTAs were identified as the predominant cause of spinal injuries [11,14,15]. In contrast, other studies have documented falls as the leading mechanism of injury, particularly in rural settings and among older adults [10,13,16,17].

In the present study, the majority of patients (89.33%) sustained spinal injuries following a dangerous mechanism of injury, warranting radiological evaluation to exclude cervical spine injury according to the CCR. In contrast, only 10.7% of patients experienced non-significant mechanisms of injury that did not require diagnostic imaging based on the CCR. The CCR incorporates three high-risk criteria, five low-risk criteria, and the patient’s ability to actively rotate the neck, which collectively assist emergency physicians in determining the need for cervical spine imaging. In EDs, physicians often have a low threshold for requesting cervical spine imaging because of the serious consequences of missing a cervical spine injury. This frequently results in unnecessary radiographic examinations, increasing healthcare costs and exposing patients to avoidable radiation. In the present study, the CCR proved to be a simple and practical clinical decision tool for identifying patients who required cervical spine imaging. Although plain cervical spine radiography has traditionally been the initial imaging modality, obtaining high-quality radiographs in emergency settings may not always be feasible. Consequently, CT has emerged as a more reliable alternative because of its superior sensitivity and diagnostic accuracy for detecting cervical spine injuries [18].

Our findings showed the T10 vertebra (12%) as the most commonly injured level, followed by T7 and L5 (8% each), indicating a thoracolumbar junction vulnerability. This pattern is echoed in Muleta and Kifle [12], where the T10-L2 region accounted for 35.3% of traumatic spine injuries, underlining this anatomical transition zone as a high-risk area. In contrast, Birua et al. [13] noted a cervical predominance (52.63%), especially at C5-C6, suggesting regional variability in injury type depending on fall dynamics and occupational hazards. Wakim et al. [19], in a U.S.-based analysis of lumbar fractures, found a rising trend in L1-L2 injuries, particularly associated with ladder and stair-related falls, highlighting the mechanical susceptibility of this segment in both developing and developed settings. Collectively, our study supports the understanding that the thoracolumbar spine is structurally vulnerable to flexion-compression and rotational forces, especially in high-velocity trauma.

According to the ASIA classification, most patients were graded D (38.6%) and C (25.3%), representing incomplete motor injuries, with fewer patients showing complete deficits (Grades A and B) or normal function (Grade E). Moshi et al. [10] also reported a similar finding, with the majority of their patients having incomplete cord injuries, although complete tetraplegia was not uncommon among cervical trauma cases. In Ethiopia, Muleta and Kifle [12] found that 17.4% of patients had complete neurological deficits, predominantly in rotational (type C) injuries, matching our pattern of incomplete injury predominance but showing a higher risk with certain CT patterns. Thus, incomplete injuries (ASIA C and D) are the most common presentations, offering a relatively better functional prognosis compared to complete spinal cord injuries.

Despite classifying 67 patients as high-risk mechanisms of injury according to the CCR, only 30 (44.7%) patients were confirmed to have cervical spine injuries. This finding suggests that while the CCR demonstrates excellent sensitivity for identifying patients at risk, it may overestimate the likelihood of cervical spine injury, resulting in a considerable number of false-positive classifications. However, such overestimation is acceptable in emergency trauma care, where maximizing sensitivity and minimizing missed cervical spine injuries are prioritized over specificity.

Surgical intervention in patients with traumatic spinal cord injury is primarily indicated to relieve neural compression, restore spinal stability and alignment, prevent further neurological deterioration, and facilitate early mobilization and rehabilitation. The most important indication is the presence of progressive or significant neurological deficits associated with radiological evidence of spinal cord or nerve root compression caused by bone fragments, intervertebral disc herniation, epidural hematoma, or vertebral displacement. Surgery is also recommended in patients with mechanically unstable spinal injuries, including fracture-dislocations, unstable burst fractures, and injuries involving disruption of the ligamentous complex, as these carry a high risk of further spinal cord damage and deformity if left untreated. Patients who fail conservative management because of worsening deformity, instability, or persistent symptoms may also require operative treatment. Current evidence further supports early surgical decompression, preferably within 24 hours of injury whenever feasible, particularly in patients with incomplete spinal cord injuries, as it has been associated with improved neurological recovery, shorter hospital stay, earlier rehabilitation, and a lower incidence of secondary complications such as pressure ulcers, respiratory infections, and venous thromboembolism [20]. Although selected patients with stable fractures and no neurological deficit may be managed conservatively, surgical stabilization remains the standard of care for unstable spinal injuries or those associated with neurological impairment. In our study, surgical treatment was needed for 56%, while 44% were managed conservatively, indicating a balanced approach based on injury severity and resource availability. Therefore, the decision to operate should be individualized based on neurological status, spinal stability, imaging findings, associated injuries, and the patient’s overall medical condition, following established evidence-based clinical guidelines.

Complications documented in our study included deep vein thrombosis (13.3%), pressure ulcers (14.7%), urinary tract infection (13.3%), and pneumonia (13.3%), while 26.7% had no complications, and 18.7% of patients had multiple complications. Multiple complications were seen more often among those with premorbid conditions such as diabetes, osteoporosis, cardiovascular diseases, and other pre-existing diseases. Most complications were mainly due to immobilization and neurologic deficit in spinal injury patients. Moshi et al. [10], in Tanzania, also reported pressure ulcers (19.7%) and respiratory complications (15%) as common issues, which showed a slightly greater proportion when compared to our study. Cao et al. [21] further confirmed high ED revisit and hospitalization rates among chronic spinal cord injury patients in the United States, with pneumonia and systemic infections being dominant causes, particularly in those with longstanding paraplegia. These studies, spanning both high- and low-resource settings, affirm that spinal injury care must prioritize early mobilization, prophylactic anticoagulation, and pressure sore prevention to reduce complications and enhance recovery.

Our study observed a mortality rate of 26.7%, with 73.3% surviving until discharge. This high mortality is consistent with global data from settings where trauma severity and limited prehospital care delay definitive management. Studies by Lalwani et al. [22] and Selvarajah et al. [23] have also reported a similar mortality rate. A high mortality rate emphasizes the urgent need for rapid referral, early decompression, and intensive care support to reduce preventable deaths in spinal trauma. Risk factors for mortality in our study were those aged more than 40 years, cases with injury at the cervical level, ASIA scale score (A and B), and the presence of complications. In the study by Sreeharsha et. al. [24], associated chest injuries (p = 0.0001), cervical spine injury (p = 0.0001), and ASIA-A neurology (p < 0.01) were found to be correlated with mortality. The risk of mortality is higher with the increasing degree of neurologic impairment. Highest mortality has been documented among those with high cervical injuries, with a gradual reduction in mortality with lower levels of injury. DiMarco et al. [25] found higher age, male sex, and greater force of injury to be associated with increased risk for mortality.

The present study has attempted to evaluate the etiology, anatomical distribution, and severity of spinal injuries, providing valuable insights into the epidemiology of spinal trauma in a tertiary care setting. As it was conducted prospectively, recall bias was minimized with better documentation of patients’ characteristics. A follow-up assessment was conducted for a month, and we have evaluated the proportion with early recovery and those with worsening of symptoms. Lastly, we have also identified factors associated with a greater risk of mortality.

Limitations

Despite its strengths, this study has certain limitations. The sample size, though adequate for descriptive analysis, limits the generalizability of results across broader populations with diverse trauma patterns. Being a single-center study, findings may reflect regional healthcare practices and referral biases rather than national trends. Long-term functional outcomes beyond hospital discharge were not assessed, restricting insight into chronic disability and rehabilitation impact. Furthermore, while radiological and clinical parameters were rigorously recorded, advanced imaging (MRI) and neurophysiological monitoring were not performed, which may have affected classification accuracy in some cases. Future multicentric studies with larger cohorts and long-term follow-up are needed to validate these findings and inform standardized spinal injury care pathways.

## Conclusions

This prospective observational study comprehensively evaluated the clinical, radiological, and functional status of patients with spinal injuries, providing insight into the epidemiology and management trends in our tertiary care setting. The findings reveal that spinal trauma predominantly affects middle-aged adults, with a slight male predominance and common mechanisms including RTAs and falls, primarily resulting in thoracolumbar injuries. A significant proportion of patients presented with neurological deficits, most commonly incomplete injuries, with wedge compression fractures and canal compromise frequently observed on imaging. Surgical management, particularly decompression and fixation, demonstrated neurological improvement in select cases. High risk for mortality were those with higher age (>40 years) and having a higher level of spinal cord injury with an ASIA score of A or B, which needs urgent attention in the ED.
